# Relationship between Neural Rhythm Generation Disorders and Physical Disabilities in Parkinson’s Disease Patients’ Walking

**DOI:** 10.1371/journal.pone.0112952

**Published:** 2014-11-11

**Authors:** Leo Ota, Hirotaka Uchitomi, Ken-ichiro Ogawa, Satoshi Orimo, Yoshihiro Miyake

**Affiliations:** 1 Department of Computational Intelligence and Systems Science, Tokyo Institute of Technology, Yokohama, Kanagawa, Japan; 2 Department of Neurology, Kanto Central Hospital, Setagaya, Tokyo, Japan; Hospital General Dr. Manuel Gea González, Mexico

## Abstract

Walking is generated by the interaction between neural rhythmic and physical activities. In fact, Parkinson’s disease (PD), which is an example of disease, causes not only neural rhythm generation disorders but also physical disabilities. However, the relationship between neural rhythm generation disorders and physical disabilities has not been determined. The aim of this study was to identify the mechanism of gait rhythm generation. In former research, neural rhythm generation disorders in PD patients’ walking were characterized by stride intervals, which are more variable and fluctuate randomly. The variability and fluctuation property were quantified using the coefficient of variation (CV) and scaling exponent α. Conversely, because walking is a dynamic process, postural reflex disorder (PRD) is considered the best way to estimate physical disabilities in walking. Therefore, we classified the severity of PRD using CV and α. Specifically, PD patients and healthy elderly were classified into three groups: no-PRD, mild-PRD, and obvious-PRD. We compared the contributions of CV and α to the accuracy of this classification. In this study, 45 PD patients and 17 healthy elderly people walked 200 m. The severity of PRD was determined using the modified Hoehn–Yahr scale (mH-Y). People with mH-Y scores of 2.5 and 3 had mild-PRD and obvious-PRD, respectively. As a result, CV differentiated no-PRD from PRD, indicating the correlation between CV and PRD. Considering that PRD is independent of neural rhythm generation, this result suggests the existence of feedback process from physical activities to neural rhythmic activities. Moreover, α differentiated obvious-PRD from mild-PRD. Considering α reflects the intensity of interaction between factors, this result suggests the change of the interaction. Therefore, the interaction between neural rhythmic and physical activities is thought to plays an important role for gait rhythm generation. These characteristics have potential to evaluate the symptoms of PD.

## Introduction

Walking is one of the most fundamental factors in our daily behaviors. The gait dynamics is thought to be generated by the interaction between neural rhythmic activity and physical activity [1], [2]. However, because this interaction is difficult to estimate in healthy gait dynamics, we focused on the patients with Parkinson’s disease (PD) (as a typical example of neurodegenerative disease) [3], which causes not only neural rhythm generation disorders, but also physical disabilities. To identify the mechanism of gait rhythm generation, we attempted to examine the relationship between neural rhythmic activity and physical activity in PD patients.

In previous studies, two symptoms were reported as neural rhythm generation disorders in PD patients’ walking. One symptom was the increase in the variability of gait rhythm [4], [5], and the other symptom was a change in the fluctuation property of gait rhythm from the normal 1/*f-*like fluctuation property [6]–[8]. In healthy young people, gait rhythm is not constant; rather, it changes subtly. This change can be described by a pair of physical measures. One is the coefficient of variation (CV), which represents the variability of gait rhythm. The other is the scaling exponent α, which represents the fluctuation property of gait rhythm and can be calculated by detrended fluctuation analysis (DFA). In particular, the fluctuation in gait rhythm is an important feature of walking. In healthy young people, the gait rhythm exhibits small variation and 1/*f*-like fluctuation properties [9].

For each of these symptoms, two types of gait rehabilitation methods using sensory cues have been proposed. One is gait training with rhythmic stimuli, which is based on forced entrainment for human, including rhythmic auditory stimulation (RAS) gait training [10] and treadmill training [11]. The other is gait training with rhythmic stimuli, which is based on mutual entrainment with human, such as WalkMate gait training [12]. In RAS gait training, fixed-tempo rhythmic auditory stimuli are input to PD patients [10]. This type of rehabilitation improves mainly the variability of gait rhythm. In other words, RAS gait training decreases CV but does not change α much [8], [13]. We have been developing the WalkMate system [12]. In WalkMate gait training, rhythmic auditory stimuli mutually entrained with the gait rhythm of PD patients [14]. This type of rehabilitation improves mainly the fluctuation property of gait rhythm [14]. In one study, α improved substantially, but CV did not change much after four consecutive days of WalkMate gait training [15]. These findings suggest that RAS gait training and WalkMate gait training improve different features of neural rhythm generation disorders in PD patients’ walking.

Conversely, PD patients often also show physical disabilities. Postural instability is one of the main motor symptoms of physical disability in PD, and its clinical manifestations are a festinant and shuffling gait, poor postural alignment, and defective postural reflexes. There are many tests for assessing the postural instability and balance control related to the risk of falling [16]. Regarding gait dynamics, the pull test [17], which is a test of postural reflex disorder (PRD), is the most suitable to evaluate physical disabilities during a dynamic state, such as walking.

However, it is not clear whether CV and α, which evaluates the different features of neural rhythm generation disorders, are related to PRD, which evaluate physical disabilities. The purpose of this study was to examine the relationship between the set of CV and α, and PRD on a platform aimed at evaluating the gait rhythm in PD patients, to identify the mechanism of gait rhythm generation. To construct this evaluation platform, we focused on a combination of CV and α, because it can be considered as a feature amount that represents neural rhythm generation disorders. Subsequently, we classified the subjects according to the presence or absence of PRD using the platform for gait rhythm. Furthermore, the severity of PRD in a group of PD patients was classified using the platform for gait rhythm. The modified Hoehn–Yahr (mH-Y) scale [18], [19] was used as the method of evaluation of the clinical signs of PRD.

In the Methods section, we describe the demographic information of participants, gait task, and the method of measurement of stride interval. Then, we explain the calculation of two dynamic indicators: the variability of the stride interval (CV) and the fluctuation property of the stride interval (α). We mention a linear discriminant analysis using a combination of CV and α, and the classification of PRD using mH-Y. In the Results section, the results of the two classifications are shown, and the accuracy and contribution of CV or α to the classifications are reported. In the Discussion section, we discuss the mechanism underlying neural rhythm generation disorders in PD patients’ walking and a potential application of this platform to evaluate the motor symptoms of PD.

## Methods

### Participants

Forty-five patients (21 men, 24 women; mean age ± SD, 69.8±8.2 years) with PD and 17 age-matched healthy people (10 men, seven women; mean age, 70.2±2.8 years) participated in this study (Table 1). The mean disease duration (± SD) was 4.7±3.9 years. The mH-Y classifications and number of subjects were mH-Y 1–2 (*n* = 19), mH-Y 2.5 (*n* = 11), and mH-Y 3 (*n* = 15). All patients were taking at least one of antiparkinsonian medications during the experiment. The antiparkinsonian medications included levodopa/carbidopa, dopamine receptor agonist, selegiline, amantadine, and anticholinergics. Those were taken at maximum two hours before the start time of measurement. All participants could walk without a cane or walker. These experimental procedures were approved by the Kanto Central Hospital Ethics Committee. Before the experiment, we obtained written informed consent from the participants.

**Table 1:** Characteristics of the participants.

Classification	Difference between PRD and no-PRD			Difference between obvious-PRD and mild-PRD		
Positive/Negative	Positive (PRD, *n* = 26)	Negative (no-PRD, *n* = 36)	*P*	Positive (obvious-PRD, *n* = 15)	Negative (mild-PRD, *n* = 11)	*P*
Age (years, mean ± SD)	72.7±7.0	68.1±6.9	0.01	72.5±6.7	72.4±7.5	0.83
Sex (male:female)	15∶11	16∶20	0.31	8∶7	7∶4	0.61
Disease duration (years, mean ± SD)	4.9±4.6	2.3±3.1	0.01	6.2±5.5	4.0±2.8	0.35
mH-Y score in “on” state (median, range)	3, 2.5–3	1.25, 0–2	–	3, 3	2.5, 2.5	–

*P* values were calculated using Welch’s two-sample *t* test.

PRD, postural reflex disorder; mH-Y, modified Hoehn–Yahr scale.

### Gait tasks and measurement of stride interval

Participants walked at their preferred pace along a 200 m round course. We measured gait rhythm once for each participant and calculated the stride interval time series. Stride interval is defined as the time duration between two consecutive foot contacts on the same side. Foot switches (OT-21BP-G, Ojiden, Japan) were attached under the shoes and were used to detect the gait rhythm. The mean number (± SD) of stride intervals was 154±23 strides for the 200 m. Data for foot contact timing were sent to a laptop PC (CF-W5AWDBJR, Panasonic, Japan) via a wireless transmitter (S-1019M1F, Smart Sensor Technology, Japan). The sampling frequency was 100 Hz. We used only the data obtained for the left side because no significant differences between stride interval were observed between the left and right sides (left side: mean = 1.06±0.09 s, CV = 2.73% ±1.09%, α = 0.80±0.21; right side: mean = 1.06±0.09 s, CV = 2.78% ±1.62%, α = 0.81±0.22; *P-*values based on Welch’s two-sample *t* test: *P* = 0.97 for mean, *P* = 0.82 for CV, *P* = 0.92 for α). We analyzed the data obtained for the right side in only one patient because a high noise level was observed in the data for the left side. To assess only the stable stride interval phase, the first 10 strides and last five strides (i.e., the transient stride interval phase) were not analyzed.

### CV

We focused on the CV as a dynamic indicator to evaluate the variability of stride interval in the participants. CV represents the variability of time-series data, and is calculated as the standard deviation normalized to the mean value: CV = SD/Mean×100 [%]. The CV of healthy people is 1%–2.5%, and the CV of PD patients is 2.5%–4% [6].

### DFA

We focused on the scaling exponent α as the other dynamic indicator to evaluate the fluctuation property. The scaling exponent α can be quantified by DFA as a long-range correlation in time series data [20], [21]. We selected this method because it can also be applied to relatively short intervals [22].

If the α is nearly equal to 0.5, the time series is characterized by white noise. On the other hand, if α is near 1.0, the series is characterized by 1/*f* fluctuation and is suggested to be generated by chaos dynamics or limit cycle dynamics coupled with noise [23]–[26]. The α of the stride interval at the preferred pace has been reported as 0.50–0.85 in PD patients [6], [8] and as 0.8–1.2 in healthy young people [27], [28]. In healthy elderly people, the α of the stride interval is decreased to 0.7–0.9, although the CV remains unchanged [7], [28], [29].

### Linear discriminant analysis

Fisher’s linear discriminant analysis was used with a combination of CV and α to obtain a function for dividing the measured data into two groups [30].

The leave-one-out cross-validation method was used to estimate the classification rate, and the following were calculated: (1) accuracy, the rate of truly classified data among all data; (2) sensitivity, the accuracy rate for identifying positive data (participants with more severe symptoms); and (3) specificity, the accuracy rate for identifying negative data (participants with mild symptoms). To compare the individual contribution to the classification of CV and α, these two variables were normalized using a Z score, and the angle between the normalized CV axis and the boundary line was calculated by a linear discriminant function.

### Classification of PRD

**Figure 1:**
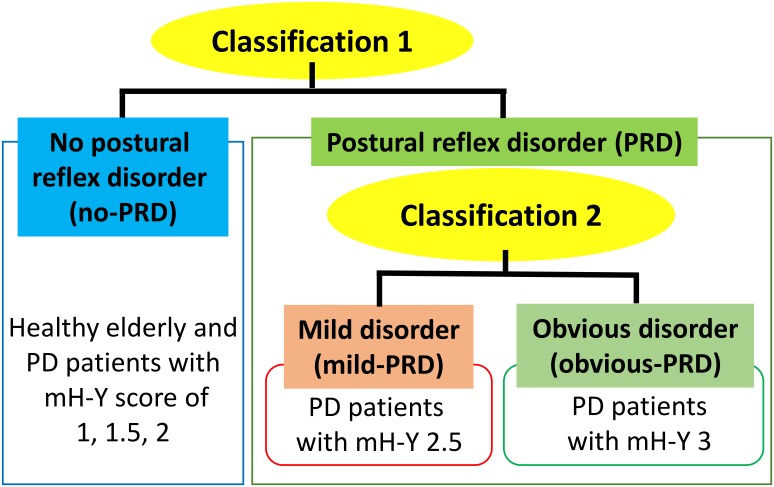
Classification of the severity of postural reflex disorder (PRD).

Walking is controlled in parallel with posture and muscle-tone control [31]–[34]. Postural instability, which is one of the physical disabilities, can be often evaluated by the Berg Balance Test [16]. However, when considering gait dynamics, the pull test (30^th^ item in the Unified Parkinson’s Disease Rating Scale) is the most suitable for estimating the ability of physical activities in walking [17]. Therefore, we focused on the pull test to identify the presence or absence of PRD and its severity. In the pull test, the shoulder of a PD patient is pulled backward and forward while the patient remains standing. An overview of the classification is shown in Figure 1. Performance on the pull test is associated with mH-Y, which is one of the clinical indicators used for the assessment of motor symptoms of PD [19].

The scores in the original H-Y range from 1 to 5, in increments of 1. The mH-Y includes added stages 1.5, and 2.5 [19]. We separated participants into three groups based on their mH-Y score and performance on the pull test: mH-Y score of 2 or less with no problems on the pull test (no-PRD), mH-Y = 2.5 with signs of mild disorder on the pull test (mild-PRD), and mH-Y = 3 with obvious signs of disorder on the pull test (obvious-PRD). No-PRD was determined by a normal postural reflex in the pull test. Mild-PRD was defined by very mild postural impairment (suggestive, but not diagnostic; usually one or two steps before recovery from a postural threat) [19]. Obvious-PRD was determined by the presence of retropulsion, which is defined by (1) the appearance of more than three backward steps during the pull test, followed by unaided recovery, (2) the absence of postural reflex, or (3) the indication of falling if the examinee is not supported [17]. Although the examiner’s decision regarding need of support in the pull test is subjective, we paid careful attention to the classification of PRD. All participants were examined by the same doctor in the same environment. Furthermore, the doctor is a PD expert who is authorized by the Japanese Society of Neurology. Therefore, the results of the mH-Y staging were reproducible.

We first classified participants according to the presence and absence of PRD (Classification 1 in Figure 1, see Table 1). This classification segregated the no-PRD group (17 healthy elderly people and two PD patients, one with an mH-Y score of 1 and one with a score of 1.5) from the PRD group. We then divided the PRD group of patients into the mild-PRD and obvious-PRD groups (Classification 2 in Figure 1, see Table 1).

## Results

**Figure 2:**
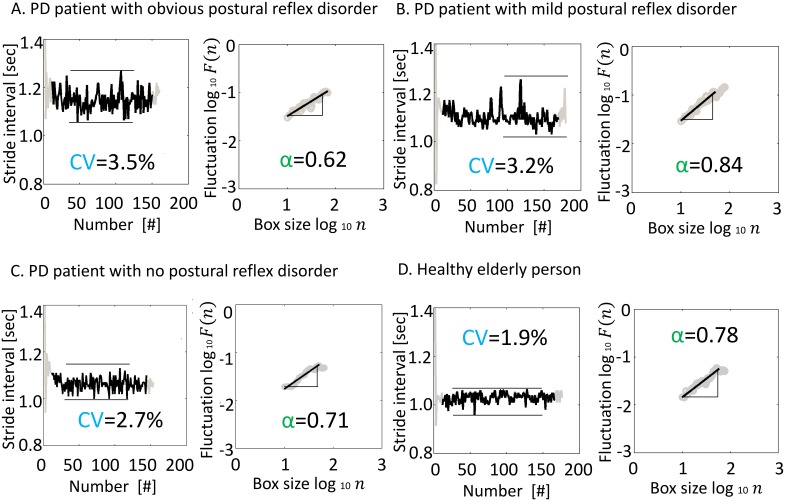
A sample of the stride interval and the fluctuation relative to box size. (A) PD patient with obvious postural reflex disorder (mH-Y score, 3; age 76 years; male). (B) PD patient with mild postural reflex disorder (mH-Y score, 2.5; age, 70 years; male). (C) PD patient with no postural reflex disorder (mH-Y score, 2; age, 76 years; male). (D) Healthy elderly person (age. 71 years; male).

Figure 2 shows a sample result of the gait analysis, including the stride interval time series and the result of DFA. The CV of the stride interval was larger in PD patients with PRD (Figure 2A, B) than in healthy people (Figure 2C). The α of the stride interval (Figure 2A) was lower in PD patients with obvious-PRD than in PD patients with mild-PRD (Figure 2B), in PD patients with no-PRD (Figure 2C), or in healthy elderly people (Figure 2D).

### Classification 1: The presence or absence of PRD

**Figure 3:**
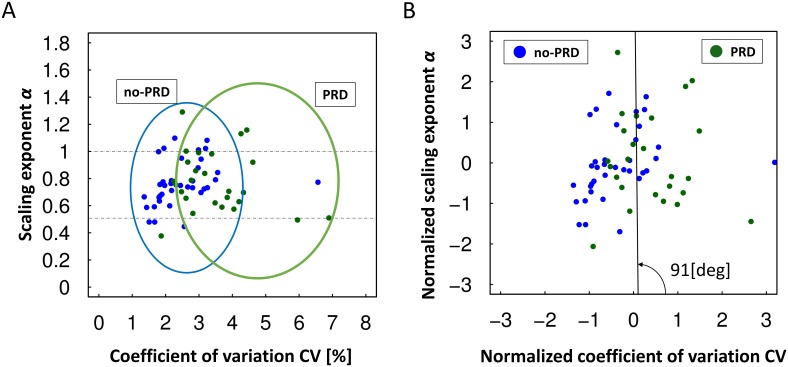
Classification according to the presence or absence of postural reflex disorder. The no postural reflex disorder group (no-PRD) is indicated by blue points, and the postural reflex disorder group (PRD) is indicated by green points. The x-axis represents the CV, and the y-axis represents α. The data for the no-PRD group are distributed in a small CV region around 2%, whereas those for the PRD group are distributed in a large CV region roughly from 2.5% to 5%. The two groups have a wide and overlapping distribution of α. (A) Distribution of the original data. (B) Distribution of the normalized data. The solid line represents the boundary between the no-PRD group and the PRD group.

First, we classified the PD patients and healthy elderly people into two groups according to the presence or absence of PRD. The no-PRD group comprised healthy elderly participants and PD patients with an mH-Y score of 1–2, and the PRD group comprised PD patients with an mH-Y score of 2.5–3. Figure 3A shows the distribution of all participants’ data for the feature space configured by CV and α of the stride interval; namely, (CV, α) plane. The x-axis represents CV, and the y-axis represents α. The blue points represent the data for the no-PRD group, and the green points represent the data for the PRD group. On the y-axis of α in Figure 3A, the data for each group overlapped between 0.5 and 1.0. In contrast, the no-PRD group data were distributed in a scattered pattern in the low-CV area, and the data for the PRD group were scattered in the high-CV area.

Figure 3B shows the distribution of normalized data, to indicate which axis contributes to the classification of the two groups regardless of the variation in each axis. The solid line represents the boundary line between the two groups. When we defined the no-PRD group as negative and the PRD group as positive, the accuracy was 74%, the sensitivity was 50%, and the specificity was 92%. The angle between the normalized CV axis and the boundary line shown in Figure 3B was 91°. The large angle observed between the normalized CV axis and the boundary line suggests that the CV can be used to differentiate between the presence and absence of PRD.

### Classification 2: Obvious-PRD or mild-PRD

**Figure 4:**
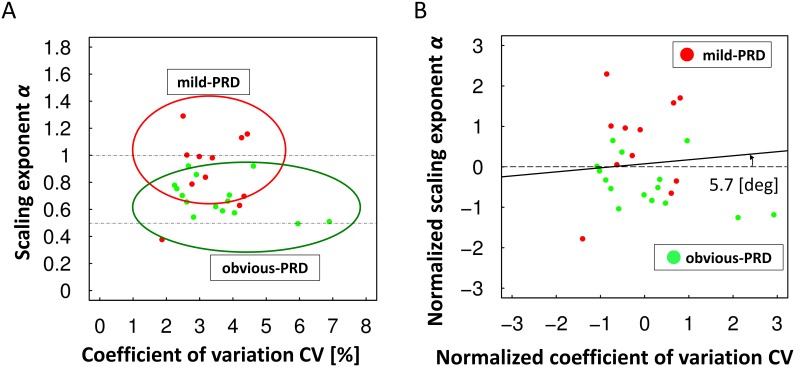
Classification of obvious and mild postural reflex disorder. The mild postural reflex disorder group (mild-PRD) is indicated by red points, and the obvious postural reflex disorder group (obvious-PRD) is indicated by light-green points. The x-axis represents the CV, and the y-axis represents α. (A) Distribution of the original data. (B) Distribution of the normalized data. The solid line represents the boundary between the mild-PRD and obvious-PRD groups.

Next, we focused on the two PRD groups: obvious-PRD and mild-PRD. The mild-PRD group comprised PD patients with an mH-Y score of 2.5, and the obvious-PRD group comprised PD patients with an mH-Y score of 3. Figure 4A shows the distribution of data of the PRD group in CV, α) plane. The red points represent the data for the mild-PRD group, and the light-green points represent the data for the obvious-PRD group. On the x-axis of CV in Figure 4A, the data for both groups overlap between 2.5 and 6.0. By contrast, the α for the mild-PRD group tended to scatter near 1.0 (i.e., the 1/*f*-like fluctuation property was observed), and the α for the obvious-PRD group tended to scatter around 0.6 (i.e., the 1/*f*-like fluctuation property was detected less often).

When we defined the mild-PRD group as negative and the obvious-PRD group as positive, the accuracy was 69%, the sensitivity was 80%, and the specificity was 55%. The solid line represents the boundary line between the two groups, and the dashed line represents a horizontal line that corresponds to the average level of the original value of α. The angle between the normalized CV axis and the boundary line shown in Figure 4B was 5.7°. The small angle observed between the normalized CV axis and the boundary line suggests that α can be used to differentiate obvious-PRD from mild-PRD.

## Discussion

In this study, we used CV to evaluate the variability of gait rhythm, and the scaling exponent α to evaluate the fluctuation property of gait rhythm. These are two important indicators of neural rhythm generation disorders in PD patients. We performed a linear discriminant analysis based on a combination of CV and α to differentiate between the presence and absence of PRD, and between obvious-PRD and mild-PRD. As a result, CV differentiated between the presence and absence of PRD, indicating the strong correlation between the change of CV and symptoms of PRD. Considering that the mechanism of PRD is independent of the neural rhythmic activities, this result suggests the existence of feedback process from physical activities to neural rhythmic activities. Furthermore, α differentiated between mild-PRD and obvious-PRD. Considering α reflects the strength of interaction or relationship between factors, this result suggests the existence of interaction between physical activities and neural rhythmic activities. Therefore, the interaction between neural rhythmic activities and physical activities is thought to play an important role for gait disabilities in PD.

**Figure 5:**
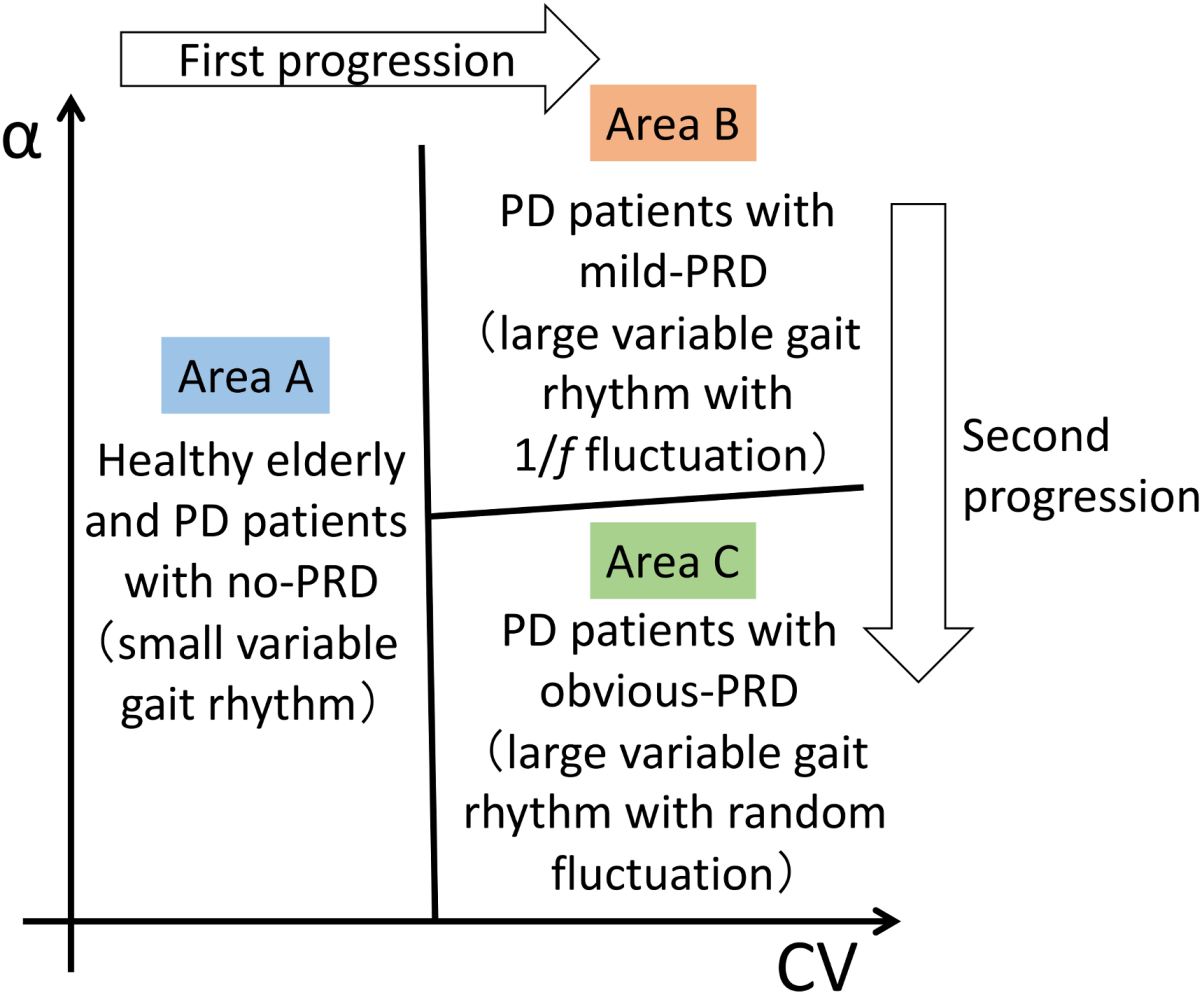
Concept of the evaluation platform. The x-axis is related to the CV of the stride interval, and the y-axis is related to the α of the stride interval. PRD, postural reflex disorder.

We now discuss the relationship between neural rhythm generation disorders and physical disabilities based on the results obtained in this study. Figure 5 summarizes the results of the classification used in this study. CV and α are dynamic indicators of neural rhythm generation disorders, and PRD is a clinical indicator of the severity of a physical disability. Area A in Figure 5 represents a low CV: i.e., the variability of the gait rhythm of the participants was small. Moreover, the participants who appeared in this area had no symptoms of PRD. Area B represents a large CV and a high α: i.e., the variability was large but the 1/*f*-like fluctuation property was observed. In addition, these patients had mild symptoms of PRD. Area C represents a large CV and a low α: i.e., the variability of the gait rhythm of the patients was large, and the 1/*f*-like fluctuation property was not observed. In addition, these patients showed obvious symptoms of PRD.

When considering the manner in which neural rhythm generation disorders progress during the transition from the healthy state to obvious-PRD, we find an important factor in walking. At first, the patient’s gait rhythm tends to transfer from the area A to area B in (CV, α) plane during the transition from no-PRD to mild-PRD. This result shows that there are strong correlation between the occurrence of PRD and the increase of CV. It might be natural to be considered that the change of neural rhythm generation give rise to the disabilities for physical activity. However the mechanism of PRD is independent of development of neural rhythmic generation, because the enhancement and suppression function of the muscle tone, which is related to the occurrence of PRD, is considered to work in parallel with the function of gait rhythm generation [31]–[34]. Therefore, this means that physical activity is fed back to neural rhythm activity in walking. Considering the fact that neural rhythmic activity always affects the physical activity, our result suggests the existence of interaction between neural rhythmic activity and physical activity. Next, the gait rhythm tends to transfer from area B to area C during the transition from mild-PRD to PRD. This shows that there are strong correlation between progression of the severity of PRD and the decrease of α. In other words, this transition is observed as the weakening process of the 1/*f* fluctuation from the state we can observe the 1/*f* fluctuation. The 1/*f* fluctuation property is defined by the frequency spectrum whose power is proportional to the inverse of frequency. In general, 1/*f*-like fluctuation is mainly generated by the interaction between multiple factors [9], [23]–[28]. Therefore, the change of time series structure of the gait rhythm can be regarded as the intensity change of interaction. This result suggests that the intensity of interaction on the mechanism of gait rhythm generation is weakened by the progression of severity of PRD. This complement the fact that the interaction between neural rhythmic activity and physical activity plays an important role in gait rhythm generation.

When considering all these points, we summarize the development mechanism of gait rhythm generation disorders. The gait rhythm of the patients in area A (small CV) in Figure 5 represented the state without physical disabilities. The gait rhythm of the patients in area B (large CV, high α) represented the state that is controlled mainly by the interaction between neural rhythmic and physical activities against the physical disabilities. The gait rhythm of the patients in area C (large CV, low α) represented the destabilized state in response to weakening of the interaction between neural rhythmic and physical activities. These findings suggest that it is possible to construct an evaluation platform for neural rhythm generation disorders by combining the CV and α parameters, and to use this system to evaluate the progression of physical disabilities.

This information on Figure 5 may provide clues for evaluating the progress of PD patients during rehabilitation using RAS gait training or WalkMate gait training. RAS gait training decreases CV [8], [13]. This type of gait training may improve the first progression involving variability of gait rhythm: i.e., transition from the right half-plane (area B or area C) to the left half-plane (area A) in (CV, α) plane. On the other hand, the WalkMate gait training increases the 1/*f*-like fluctuation property [14], [15]. This type of gait training may improve the second progression involving the fluctuation property: i.e., the transition from area C to area B. Therefore, based on this platform, we were able to extract information not only on the presence or absence of PRD, but also on the severity of PRD using (CV, α) plane for evaluating neural rhythm generation disorders. Using this system to evaluate neural rhythm generation disorders may help physical therapists to choose a rehabilitation method that fits the severity of the patients’ physical disabilities.

## References

[1] Pearson KG (2000) Neural adaptation in the generation of rhythmic behavior. Annu Rev Physiol 62: 723–753. 10845109 10.1146/annurev.physiol.62.1.723

[2] Taga G, Yamaguchi Y, Shimizu H (1991) Self-organized control of bipedal locomotion by neural oscillators in unpredictable environment. Biol Cybern 65: 147–159. 1912008 10.1007/BF00198086

[3] Jankovic J (2008) Parkinson’s disease: clinical features and diagnosis. J Neurol Neurosurg Psychiatry 79: 368–376. 18344392 10.1136/jnnp.2007.131045

[4] Hausdorff JM, Cudkowicz ME, Firtion R, Wei JY, Goldberger AL (1998) Gait variability and basal ganglia disorders: stride-to-stride variations of gait cycle timing in Parkinson’s disease and Huntington’s disease. Mov Disord 13: 428–437. 9613733 10.1002/mds.870130310

[5] Schaafsma JD, Giladi N, Balash Y, Bartels AL, Gurevich T, et al (2003) Gait dynamics in Parkinson’s disease: relationship to Parkinsonian features, falls and response to levodopa. J Neurol Sci 212: 47–53. 12809998 10.1016/s0022-510x(03)00104-7

[6] Hausdorff JM, Lertratanakul A, Cudkowicz ME, Peterson AL, Kaliton D, et al (2000) Dynamic markers of altered gait rhythm in amyotrophic lateral sclerosis. J Appl Physiol 88: 2045–2053. 10846017 10.1152/jappl.2000.88.6.2045

[7] Hausdorff JM (2007) Gait dynamics, fractals and falls: finding meaning in the stride-to-stride fluctuations of human walking. Hum Mov Sci 26: 555–589. 17618701 10.1016/j.humov.2007.05.003 PMC2267927

[8] Hausdorff JM (2009) Gait dynamics in Parkinson’s disease: common and distinct behavior among stride length, gait variability, and fractal-like scaling. Chaos 19: 026113 doi:10.1063/1.3147408 19566273 10.1063/1.3147408 PMC2719464

[9] Hausdorff JM, Peng CK, Ladin Z, Wei JY, Goldberger AL (1995) Is walking a random walk? Evidence for long-range correlations in stride interval of human gait. J Appl Physiol 78: 349–358. 7713836 10.1152/jappl.1995.78.1.349

[10] Thaut MH, Abiru M (2010) Rhythmic auditory stimulation in rehabilitation of movement disorders: a review of the current research. Music Percept 27: 263–269.

[11] Rubinstein TC, Giladi N, Haausdorff JM (2002) The power of cueing to circumvent dopamine deficits: a review of physical therapy treatment of gait disturbances in Parkinson’s disease. Mov Disord 17: 1148–1160. 12465051 10.1002/mds.10259

[12] Miyake Y (2009) Interpersonal synchronization of body motion and the Walk-Mate walking support robot. Robot IEEE Trans 25: 638–644.

[13] Hausdorff JM, Lowenthal J, Herman T, Gruendlinger L, Peretz C, et al (2007) Rhythmic auditory stimulation modulates gait variability in Parkinson’s disease. Eur J Neurosci 26: 2369–2375. 17953624 10.1111/j.1460-9568.2007.05810.x

[14] Hove MJ, Suzuki K, Uchitomi H, Orimo S, Miyake Y (2012) Interactive rhythmic auditory stimulation reinstates natural 1/*f* timing in gait of Parkinson’s patients. PLoS ONE 7: e32600. 22396783 10.1371/journal.pone.0032600 PMC3292577

[15] Uchitomi H, Ota L, Ogawa K-i, Orimo S, Miyake Y (2013) Interactive rhythmic cue facilitates gait relearning in patients with Parkinson’s disease. PLoS ONE 8: e72176. 24098631 10.1371/journal.pone.0072176 PMC3787045

[16] Thorbahn LDB, Newton RA (1996) Use of the Berg Balance Test to predict falls in elderly persons. Phys Ther 76: 576–583. 8650273 10.1093/ptj/76.6.576

[17] Bloem BR, Beckley DJ, Van Hilten BJ, Roos RAC (1998) Clinimetrics of postural instability in Parkinson’s disease. J Neurol 245: 669–673. 9776467 10.1007/s004150050265

[18] Hoehn MM, Yahr MD (1967) Parkinsonism: onset, progression, and mortality. Neurology 17: 427–442. 6067254 10.1212/wnl.17.5.427

[19] Goetz CG, Poewe W, Rascol O, Sampaio C, Stebbins GT, et al (2004) Movement Disorder Society Task Force report on the Hoehn and Yahr staging scale: status and recommendations. Mov Disord 19: 1020–1028. 15372591 10.1002/mds.20213

[20] Peng C-K, Buldyrev SV, Havlin S, Simons M, Stanley HE, et al (1994) Mosaic organization of DNA nucleotides. Phys Rev E Stat Phys Plasmas Fluids Relat Interdiscip Topics 49: 1685–1689. 9961383 10.1103/physreve.49.1685

[21] Peng C-K, Havlin S, Stanley HE, Goldberger AL (1995) Quantification of scaling exponents and crossover phenomena in nonstationary heartbeat time series. Chaos 5: 82–87. 11538314 10.1063/1.166141

[22] Delignieres D, Ramdani S, Lemoine L, Torre K, Fortes M, et al (2006) Fractal analyses for ‘short’ time series: a re-assessment of classical methods. J Math Psychol 50: 525–544.

[23] Goldberger AL, Amaral LA, Hausdorff JM, Ivanov PC, Peng CK, et al (2002) Fractal dynamics in physiology: alterations with disease and aging. Proc Natl Acad Sci U S A 99: 2466–2472. 11875196 10.1073/pnas.012579499 PMC128562

[24] Gates DH, Su JL, Dingwell JB (2007) Possible biomechanical origins of the long-range correlations in stride intervals of walking. Physica A 380: 259–270. 18335005 10.1016/j.physa.2007.02.061 PMC2266876

[25] Ivanov PCh, Ma QDY, Bartsch RP, Hausdorff JM, Amaral LAN, et al (2009) Levels of complexity in scale-invariant neural signals. Phys Rev E Stat Nonlin Soft Matter Phys 79: 041920. 19518269 10.1103/PhysRevE.79.041920 PMC6653582

[26] Ahn J, Hogan N (2013) Long-range correlations in stride intervals may emerge from non-chaotic walking dynamics. PLoS ONE 8: e73239. 24086274 10.1371/journal.pone.0073239 PMC3781160

[27] Hausdorff JM, Purdon PL, Peng CK, Ladin Z, Wei JY, et al (1996) Fractal dynamics of human gait: stability of long-range correlations in stride interval fluctuation. J Appl Physiol 80: 1448–1457. 8727526 10.1152/jappl.1996.80.5.1448

[28] Hausdorff JM, Ashkenazy Y, Peng C-K, Ivanov PC, Stanley HE, et al (2001) When human walking becomes random walking: fractal analysis and modeling of gait rhythm fluctuations. Physica 302: 138–147. 10.1016/s0378-4371(01)00460-5 12033228

[29] Hausdorff JM, Mitchell SL, Firtion R, Peng CK, Cudkowicz ME, et al (1997) Altered fractal dynamics of gait: reduced stride-interval correlations with aging and Huntington’s disease. J Appl Physiol 82: 262–269. 9029225 10.1152/jappl.1997.82.1.262

[30] Duda RO, Hart PE, Stork DG (2001) Pattern classification. 2^nd^ ed. New York: Wiley. 654 p.

[31] Takakusaki K, Hanaguchi T, Ohtinata-Sugimoto J, Saitoh K, Sakamoto T (2003) Basal ganglia efferents to the brainstem centers controlling postural muscle tone and locomotion: a new concept for understanding motor disorders in basal ganglia dysfunction. Neuroscience 119: 293–308. 12763089 10.1016/s0306-4522(03)00095-2

[32] Takakusaki K, Saitoh K, Harada H, Kashiwayanagi M (2004) Role of basal ganglia-brainstem pathways in the control of motor behaviors. Neurosci Res 50: 137–151. 15380321 10.1016/j.neures.2004.06.015

[33] Tomita N, Yano M (2007) Bipedal robot controlled by the basal ganglia and brainstem systems adjusting to indefinite environment. Proc 2007 IEEE/ICME International Conference on Complex Medical Engineering: 116–121.

[34] Takakusaki K, Tomita N, Yano M (2008) Substrates for normal gait and pathophysiology of gait disturbances with respect to the basal ganglia dysfunction. J Neurol 255[Suppl 4]: 19–29. 10.1007/s00415-008-4004-7 18821082

